# Developing and Implementing Provider-Training and Evidence-Based Tools to Support Pre-exposure Prophylaxis (PrEP) Decision-Making and Increase PrEP Adherence Among Young Men Who Have Sex With Men: Protocol for the PrEP Choice Longitudinal Cohort Study

**DOI:** 10.2196/64186

**Published:** 2025-03-20

**Authors:** Crissi Rainer, Rebecca Schnall, Mary R Tanner, Carla A Galindo, Karen W Hoover, Sylvie Naar, Maeve Brin, Andres Martinez, Haomiao Jia, Maria Mendoza, Lisa Hightow-Weidman

**Affiliations:** 1 Institute on Digital Health and Innovation College of Nursing Florida State University Tallahassee, FL United States; 2 School of Nursing Columbia University New York, NY United States; 3 Centers for Disease Control and Prevention Atlanta, GA United States; 4 Center for Translational Behavioral Science College of Medicine Florida State University Tallahassee, FL United States; 5 FHI 360 Durham, NC United States

**Keywords:** pre-exposure prophylaxis, PrEP, PrEP cohort, young men who have sex with men, YMSM, digital health, evidence-based tools, motivational interviewing

## Abstract

**Background:**

Despite the availability of highly effective HIV pre-exposure prophylaxis (PrEP), uptake and adherence to PrEP among young men who have sex with men (YMSM) remains low, limiting its impact on the prevention of HIV infection. Strategies that incorporate an array of prevention options and provide YMSM and their providers with tailored education and support tools, including tools to support shared decision-making, are needed.

**Objective:**

The goals of the Centers for Disease Control and Prevention (CDC)–funded PrEP Choice study include the development and deployment of CDC guideline–consistent PrEP provider training and the implementation of evidence-based provider- and client-facing PrEP education and support tools. Under this initiative, the CDC funded 2 research projects, Florida State University (the Expanding PrEP in Communities of Color [EPICC] project), and Columbia University (the mChoice project).

**Methods:**

Providers from both projects will complete the PrEP Choice online training, which was developed to educate providers on PrEP options and how to engage clients in open discussions around sexual health and PrEP options. EPICC project providers will also attend online tailored motivational interviewing (TMI) training sessions, and mChoice project providers will view a training video on cultural competency and humility in PrEP care. Following training, each project will enroll a cohort of 400 participants receiving care from study providers and follow them for 12-18 months. Participants will complete online surveys every 3 months and provide biomarkers to assess PrEP adherence. Electronic health record (EHR) data will be collected every 6 months to provide additional information on clinic attendance, PrEP prescriptions, and HIV/sexually transmitted infection (STI) testing. Each project will provide cohort participants with a unique digital health tool to support the PrEP choice and ongoing adherence. The study will assess the effectiveness of training and educational and support tools in practice and the critical factors associated with the successful uptake of and adherence to PrEP by participants. The study will also monitor patterns of PrEP use among YMSM, including types of PrEP and switching between types.

**Results:**

Formative work to develop and prepare the tools for implementation was completed in 2023. The EPICC project began provider training in early 2024, and the mChoice project began in spring 2024. Cohort enrollment for both projects began after provider training began.

**Conclusions:**

Given the changing PrEP landscape, implementation of provider education and tools to maximize uptake and adherence is needed. By delivering culturally competent and interactive provider training on PrEP options, the study will help providers counsel and guide participants on the effective and safe use of PrEP. The digital health tools created will support participant adherence to help them optimize PrEP benefits. Through the cohort design, the PrEP Choice study will provide real-world data about PrEP use that will be critical for informing future guidelines and tools.

**International Registered Report Identifier (IRRID):**

DERR1-10.2196/64186

## Introduction

### Background

Young gay, bisexual, and other young men who have sex with men (YMSM) are the population most affected by HIV in the United States. Among all HIV infections diagnosed in 2021, 67% were found in MSM [1]. Racial and ethnic disparities persist in HIV diagnoses among MSM, with 36% of diagnoses among Black or African American (Black) MSM and 33% among Hispanic or Latino (Hispanic) MSM, compared with 25% among White MSM, despite smaller overall populations of Black and Hispanic MSM compared with those of White MSM. HIV can be prevented with the use of HIV pre-exposure prophylaxis, or PrEP [2]. The US Food and Drug Administration has approved the use of 3 antiretroviral drugs for PrEP by MSM: oral emtricitabine and tenofovir disoproxil fumarate (F/TDF) in 2012, followed by the availability of generic F/TDF in 2021; oral emtricitabine and tenofovir alafenamide (F/TAF) in 2018; and long-acting injectable cabotegravir (CAB-LA) in 2021. Clinical trials of sexual event-driven PrEP regimens or 2-1-1 PrEP using F/TDF have demonstrated it to be highly effective for HIV prevention among MSM [3,4]. These options provide MSM with choices about their preferred PrEP regimen; however, little is known about the PrEP choices men make, their switching between PrEP options, and the reasons for these decisions.

PrEP coverage continues to increase markedly among men [5]. However, findings from analyses of limited race and ethnicity data for PrEP users suggest racial and ethnic disparities exist in coverage. These PrEP use disparities are especially concerning in the context of large racial and ethnic disparities in HIV diagnoses, because men who might most benefit from PrEP are not being prescribed it, and among those prescribed, adherence remains suboptimal [6-9]. To accomplish the goals of the “Ending the HIV Epidemic in the US” initiative to reduce new HIV diagnoses by 90% by 2030, increase PrEP coverage to 50% of individuals with indications, and achieve health equity [10], it is imperative to overcome racial and ethnic disparities in PrEP use.

The implementation of effective interventions is needed to increase PrEP initiation, adherence, and persistence among YMSM. YMSM need tailored education with autonomy-supportive communication related to the growing array of PrEP options, and providers who serve these clients also need education and clinical decision-making support. Implementation of resources that discuss all PrEP options and emphasize shared clinical decision-making have the potential to enhance PrEP outcomes among YMSM, including PrEP initiation, adherence, and persistence [11]. Increased understanding of PrEP use patterns, including switching among PrEP options, is needed to optimally support lifetime HIV prevention among YMSM. Using evidence-based PrEP care as a guide, provider and patient education and support tools have been developed to assist in the provision of PrEP care. Evidence-based provider and patient education and support tools (EBTs) are available but are not being routinely used in clinical settings to increase PrEP screening, counseling, initiation, adherence, and persistence by YMSM [2,12,13]. To date, there has been a lack of research on the impact that existing informational materials have on PrEP provision or how tailoring these materials to meet the needs of providers and YMSM from diverse backgrounds could enhance their effects. Further, although EBTs for both providers and YMSM are available, these resources do not acknowledge or incorporate information about a growing arsenal of prevention products nor do they maximize opportunities to engage in a shared decision-making process about these products [14,15].

Provider education can support clinicians to provide recommended PrEP services and to maintain up-to-date knowledge of PrEP options. Patient education using adapted EBTs can help men understand how PrEP can support their sexual health and learn about the PrEP options available to them. Adherence and persistence support tools, such as user-friendly apps, can support men prescribed PrEP to take their medication as directed and to continue to use it for as long as they can benefit from its protection. Increased understanding of PrEP use patterns, including switching among PrEP options, is needed to optimally support YMSM to use PrEP that is appropriate for them at various times in their lives.

### Aims and Objectives

The goals of the Center for Disease Control and Prevention (CDC)–funded study, known as the PrEP Choice study, include the development and deployment of CDC guideline–consistent PrEP provider training and the implementation of evidence-based provider- and participant-facing PrEP education and support tools [13,16]. PrEP support tools include innovative, customized mobile apps. The study will also conduct a longitudinal assessment of YMSM who are initiating or persisting with PrEP to understand their preferences for PrEP modalities and changes in PrEP use over the course of the study. The study will assess the implementation context, assess the effectiveness of provider training and EBTs in practice, and analyze the critical factors associated with successful uptake of and adherence to PrEP by participants enrolled in the study.

Under this initiative, the CDC funded 2 research projects, at Florida State University (the Expanding PrEP in Communities of Color [EPICC] project) and at Columbia University (the mChoice project). The PrEP Choice study is a 5-year initiative; funding began on September 30, 2021. In this paper, we discussed both EPICC and mChoice Studies, as they are supported under the same funding announcement, use the same PrEP training modules, use similar instruments for their longitudinal cohorts, share the same eligibility criteria, and incorporate digital health tools.

Through these 2 studies, we will test the effectiveness of provider training to increase provider knowledge of and comfort with PrEP modalities in clinical practice; evaluate the feasibility and acceptability of implementing provider training; describe the barriers and facilitators impacting the implementation of new PrEP modalities in clinical practice; test the effectiveness of provider training and digital tools to increase PrEP adherence and persistence among YMSM; and describe real-world PrEP use, including factors influencing the selection and change of PrEP regimens.

## Methods

### Ethical Considerations

This works follows a protocol reviewed and accepted by the Institutional Review Boards of Florida State University (approval number 00003623) and Columbia University (approval number AAAT8812). Participants will provide informed consent prior to beginning any study activities. The consent form includes standard consent sections describing the purpose of the study, the procedures to be followed, and the risks and benefits of participation. The consent form went through multiple reviews by team members to ensure the language was sufficiently simple. A certificate of confidentiality was obtained from the CDC. All data and records will be stored on password-protected servers. No identifiable data will be published.

EPICC project providers will be compensated US $50 for completing the pretraining survey; US $50 for completing online PrEP training modules, tailored motivational interviewing (TMI) training sessions, the posttraining survey, and the first standard patient interaction; and US $50 for the 3-month posttraining standard patient interaction. Cohort participants will be compensated US $50 for the baseline survey and app download, US $50 for an optional onboarding visit, US $25 for the 3-month follow-up survey, US $50 for the 6-month follow-up survey, US $25 for the 9-month follow-up survey, US $50 for the 12-month follow-up survey, US $25 for the 15-month follow-up survey, and US $50 for the 18-month follow-up survey. For home-based dried blood spot (DBS) collection, cohort participants will be compensated US $50 for the baseline, 6-month, and 12-month collections and US $75 for the 18-month collection. A US $50 bonus will be provided to participants who complete the first 3 collections. Participants who complete the exit interviews will be compensated US $50. Providers who complete focus group discussions will be compensated US $75.

mChoice project providers who complete the pre- and postassessment will be compensated US $50. Providers who complete an in-depth interview will be compensated US $100. Cohort participants will be compensated US $40 for the baseline survey and app download, US $45 for the 3-month follow-up survey, US $55 for the 6-month follow-up survey, US $60 for the 9-month follow-up survey, US $70 for the 12-month follow-up survey, and US $80 for the 18-month follow up survey. Cohort participants who complete an in-depth interview will be compensated US $35. Providers who complete an in-depth interview will be compensated US $100.

### Study Design

**Figure 1:**
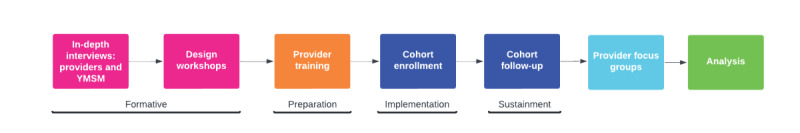
EPICC project design. EPICC: Expanding Pre-Exposure Prophylaxis (PrEP) in Communities of Color; YMSM: young men who have sex with men.

The EPICC project includes 2 distinct aims. Aim 1 includes provider training. Providers will be trained on the use of education tools adapted in the formative work and the process of screening, counseling, initiation, and follow-up with clients interested in or already taking PrEP through our PrEP training modules and will complete TMI training. The effectiveness of the training will be measured quantitatively. Aim 2 is a longitudinal cohort study designed to test the newly adapted education tools through a hybrid type 2 effectiveness implementation cohort to assess PrEP uptake and adherence to PrEP among YMSM and provider provision of PrEP and competence with education tools to increase PrEP services. Aim 2 will include a mixed methods analysis. Figure 1 provides an overview of the EPICC project’s design. Formative work can be found elsewhere [17].

**Figure 2:**
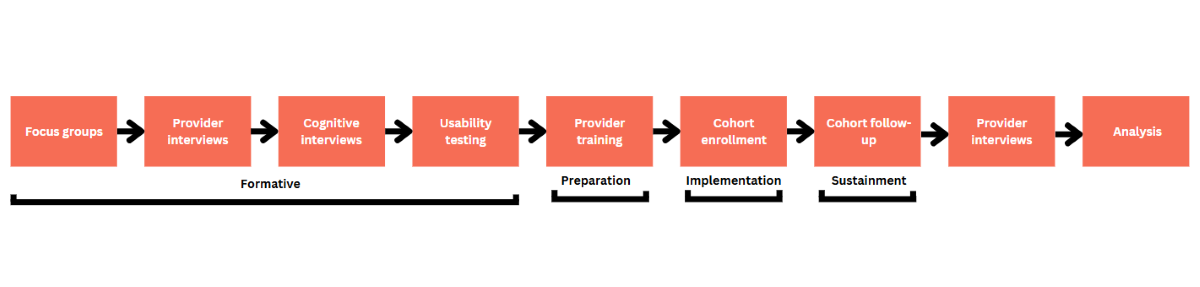
mChoice project design.

The mChoice project includes 3 aims. Aim 1 of the mChoice project is to conduct a hybrid type 2 trial testing the effectiveness of the mChoice clinical intervention to increase PrEP adherence and persistence among YMSM using PrEP. Aim 2 is to conduct in-depth interviews to assess multilevel factors at the patient level associated with selection of a PrEP regimen and switching patterns. Aim 3 is to provide training to health care providers to improve knowledge of PrEP clinical recommendations and enhance provider communication. Figure 2 provides an overview of the mChoice project design. Formative work findings can be found elsewhere [18].

### Data Collection

Every 6 months, study sites (EPICC and mChoice) will complete a clinic assessment that will include information around PrEP prescriptions issued, clinic services, financial assistance, and PrEP support services. This information will be collected prior to the provider training and will continue for the duration of the study to assess implementation efficacy. There will be a clinic assessment baseline and final, which is completed at the beginning of data collection and at the end and a shorter clinic assessment every 6 months. These assessments will capture data on clinic demographics, PrEP uptake and follow-up and provision of HIV and PrEP education.

The EPICC project’s aim 1 provider training consists of the completion of surveys prior to and after training components. Providers are rated on their use of TMI during standard patient interactions immediately after training and three months post training. Aim 2 data collection will occur using surveys every three months, project app paradata, and exit interviews. Additionally, we will conduct focus group discussions with providers at the end of the longitudinal cohort.

The mChoice project’s aim 1 data collection will occur using surveys every three months, and project app paradata. Aim 2 data collection will occur using in-depth interviews with a subsample from aim 1. The mChoice project’s aim 3 provider training consists of the completion of surveys prior to and after training components and an interview following completion of the training.

### Provider Training

**Figure 3:**
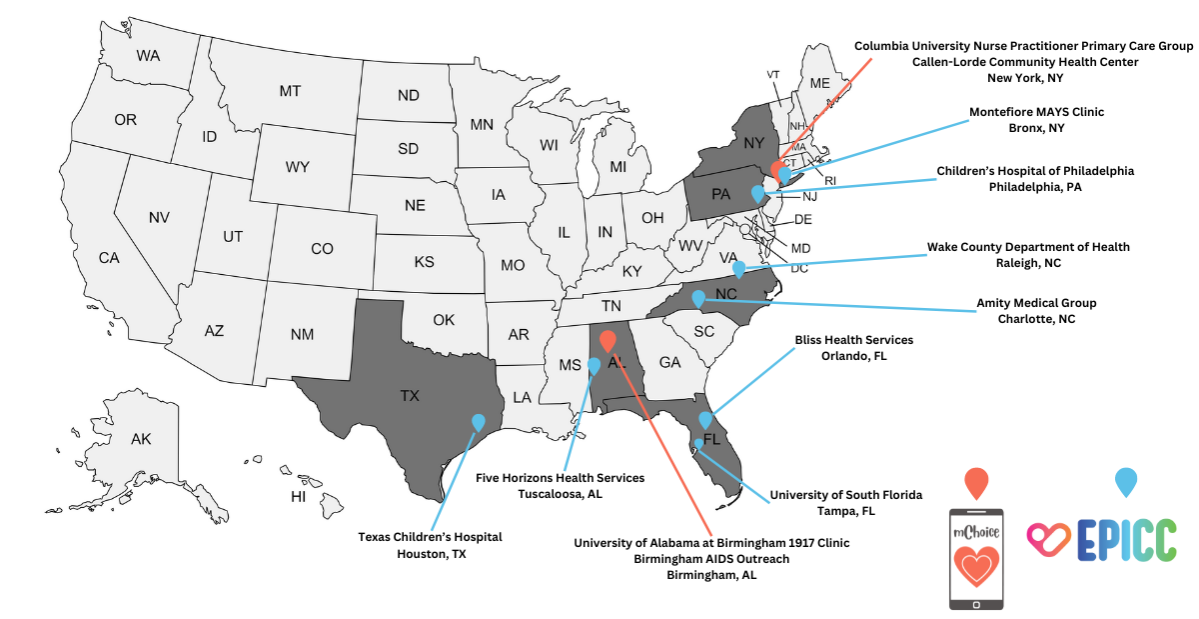
Map of EPICC and mChoice project enrollment sites. EPICC: Expanding Pre-Exposure Prophylaxis (PrEP) in Communities of Color.

Each project will enroll participants for both provider training and the cohort from unique study sites. Figure 3 includes a map of each project’s enrollment sites.

The EPICC project team developed online PrEP training modules to educate providers on currently available PrEP options and how to engage clients in open discussions around sexual health and PrEP options. The goal of these open discussions between providers and clients is to encourage shared decision-making on PrEP options. The flexible and interactive modules were created using the Easygenerator online authoring tool, which allows learners to complete their assigned courses over multiple sessions at any time. The interactive features used throughout the modules are designed to keep providers engaged. Some of these features include checks for understanding, embedded videos, and links to external content. Each module also contains at least 1 case-based scenario. These scenarios are based on real-world interactions that study team providers experience during interactions with clients.

The EPICC project team developed these modules using a variety of content sources, including CDC-produced guidelines and materials, research papers, and expert guidance. In addition to modules covering PrEP regimens, we also included modules on the need for PrEP, PrEP screening and startup, postexposure prophylaxis (PEP), and PrEP adherence and persistence. The modules were refined to their final form through an iterative feedback process with subject matter experts within the EPICC project team. Both EPICC and mChoice project teams will use these modules in their respective provider training. Table 1 lists the titles and brief descriptions of each module.

**Figure 4:**
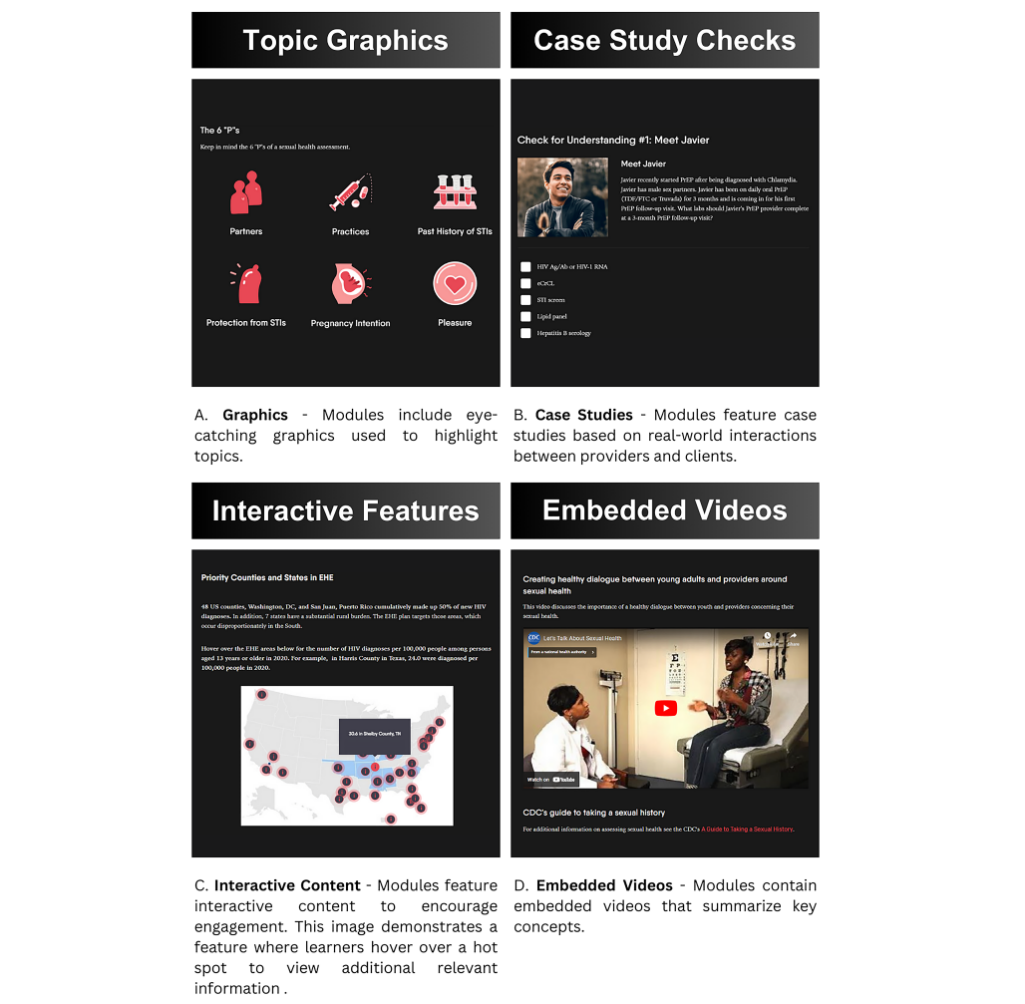
Screenshots and descriptions of interactive content within online PrEP modules used in EPICC and mChoice provider training.

Figure 4 includes screenshots of content within the modules that highlight interactive elements used throughout the course. The video image shown is from “Let’s Talk About Sexual Health” [19] produced by the CDC and BeSmartBeWell.com.

In the EPICC project, after completing the online PrEP training modules, providers will attend live, online TMI training sessions led by a member of the motivational interviewing (MI) network of trainers. The training has been split into distinct training modules: (1) introduction to TMI and PrEP choice; (2) TMI spirit, cultural humility, antiracism, and stigma reduction; (3) managing counter change talk and discord with empathy and autonomy support; and (4) eliciting and motivation for PrEP. There will be 2 training sessions lasting approximately 3 hours conducted virtually over 2 days. The training uses videos of PrEP providers demonstrating TMI integrated with antiracism and cultural humility that were created specifically for this study. The workshops are structured with cooperative learning activities, video examples, and behavioral skill acquisition steps (modeling, verbal and behavioral rehearsal, feedback). After the live, online training is completed, providers will complete a 15-minute standard patient interaction assessment, where a member of the study team will act as a client and the provider will attempt to use TMI techniques during their interaction. The conversation will be coded by a study team member using the MI Coach Rating Scale [20], and a feedback report will be generated and sent to the provider. The report will include strengths and areas for improvement, with video links featuring skills identified as areas for improvement. Three months after completing the TMI training sessions, providers will complete an additional standard patient interaction assessment with the same feedback format. Providers will also complete a brief survey prior to starting the online modules, which will include demographics; PrEP familiarity and attitudes; and PrEP use and future intentions. Providers will complete another survey after the TMI training sessions are over, which will include similar questions to the first survey to assess for change after completing online modules and TMI training.

After completing the online PrEP training modules, providers in the mChoice project will watch a 10-minute video on cultural competency and humility in PrEP care training developed specifically for this study. This module consists of an introduction to cultural competency and humility and exemplifies how these concepts are key components to PrEP care. Like the EPICC project team, sources for the video include information from CDC guidelines, CDC-produced materials, and peer-reviewed journal–cited research papers. For example, the CDC’s 5Ps approach to gathering sexual history is highlighted as a critical step in the context of PrEP care. More information about the 5Ps approach can be found on the CDC’s website [21]. Useful tips, such as using layperson’s terms that are also anatomically correct, making eye contact, and not appearing to be in a hurry, are mentioned throughout. Simultaneously, this module emphasizes the importance of making clients feel comfortable and respected and allowing the client to guide the conversation. This module aims to inform providers about how to best interact with clients and demonstrate racial and ethnic diversity among clients through the use of dynamic graphics. The module also provides training to facilitate provider-client communication so that there can be effective shared decision-making. It will be included in the PrEP Choice provider series launched through Easygenerator.

**Table 1:** Online PrEP^a^ training module titles and topics used in EPICC^b^ and mChoice Studies.

Module title	Topics covered
Module 1: Why Do We Need PrEP?	HIV epidemic in the United States, the EHE plan, PrEP update in the United States, and PrEP efficacy and effectiveness
Module 2: Who and Why for PrEP	PrEP guideline changes, PrEP screening, and HIV testing recommendations
Module 3: What Is PEP and Who Should Get It?	PEP screening, prescribing, and monitoring
Module 4: Considerations in Choosing Among Oral PrEP Options	Oral PrEP regimens, oral PrEP initiation and follow-up, 2-1-1 or on-demand PrEP, and oral PrEP side effects
Module 5: Considerations in Prescribing and Monitoring Injectable PrEP	Injectable PrEP initiation, follow-up, adherence, side effects, and future PrEP modalities
Module 6: Maximizing PrEP Adherence and Persistence	Adherence counseling components, PrEP implementation, and cost considerations

^a^PrEP: pre-exposure prophylaxis.

^b^EPICC: Expanding PrEP in Communities of Color.

### Longitudinal Cohort

Both EPICC and mChoice projects will enroll a cohort of 400 participants. Study participation will range from 12-18 months, depending on the study enrollment date. Eligibility criteria include (1) age 18-39 years, (2) the male sex, (3) ever had sex (as a top or a bottom, insertive or receptive) with a person who has a penis, (4) have an active prescription for PrEP (including both new prescriptions at baseline or refills), (5) receive care at 1 of the participating study sites, (6) provide a mailing address within the 50 states where packages can be received, (7) have daily smartphone access, and (8) be fluent in written and spoken English or Spanish. Cohort participants will be recruited by the clinic staff during scheduled visits, through study advertisements placed in clinic waiting rooms, or through study advertisements on social media platforms.

Each project team builds off prior work to adapt their unique digital health tools poised to support PrEP choice and ongoing adherence. The EPICC project builds from the HealthMpowerment (HMP) intervention. HMP is a theory-based status app designed to provide health and wellness information and resources relevant to young people via a mobile optimized platform. HMP was created based on the Integrated Behavioral Model [22-24]. HMP is flexible and easy to adapt for specific populations and health topics. The EPICC project’s version of HMP includes resources most relevant for our study population. The mChoice app was created based on the Information System Research framework and builds on formative work to develop a PrEP adherence and monitoring app [25-27]. The mChoice app was built in collaboration with Compliance Meds Technologies (CMT) CleverCap. CMT is an mHealth technology solutions provider that develops adaptable technology to promote health. The CleverCap app is linked to a CleverCap device, which is an electronic pill bottle that records when the cap opens and closes and tracks medication adherence. The CleverCap app was customized to the needs of the mChoice project to support PrEP adherence among YMSM. It offers PrEP resources, such as 2 client-facing PrEP training modules created by the study team and a sexual activity log. It can be programmed for the use of participants who are taking any of the 3 PrEP regimens: oral (daily or event driven) or injectable. Table 2 lists the features of each project’s mobile app.

**Table 2:** Description of EPICC^a^ and mChoice digital health support tools.

Feature	EPICC	mChoice
Resources	Educational content across a range of health topics, as well as support for app engagement and behavior change through information and skill building	Participant-facing PrEP training modules, links to the CDC^b^ website
Adherence support	Medication tracker to support oral, injectable, or event-driven PrEP adherence	Electronic adherence monitoring through the use of the CleverCap app and device
Sexual behavior tracking	Sexual activity log	Sexual activity log
Connection to care	“Ask the Expert” feature to allow providers to anonymously answer user questions and connect users to resources	Chat function to communicate with the study team
Engagement features	Gamification with badges and avatars earned for completing activities	Adherence statistics for participants taking oral daily PrEP
Integrations with test kits	Yes	N/A^c^

^a^EPICC: Expanding Pre-Exposure Prophylaxis (PrEP) in Communities of Color.

^b^CDC: Centers for Disease Control and Prevention.

^c^N/A: not applicable.

### Cohort Procedures

Participants will complete a computer-assisted self-interview (CASI) every 3 months while in the study. Surveys will be hosted on REDCap. EPICC surveys will be distributed through emails and links available within the study app. mChoice surveys will be completed at study sites on a tablet. The surveys will address various topics related to PrEP care engagement, PrEP usage, and PrEP adherence, as well as sociodemographics and risk factors related to PrEP adherence (Table S1 in Multimedia Appendix 1) [7,28-38].

EPICC project participants will complete a home-based DBS collection kit every 6 months while in the study to assess for levels of tenofovir-diphosphate (TFV-DP) and emtricitabine-triphosphate (FTC-TP). Additionally, EPICC project participants can attend an optional virtual onboarding visit with a member of the study team to review key features of the study app and DBS collection instructions. mChoice project participants who report using PrEP containing TFV and FTC will provide a urine sample of 15-30 mL at each visit to measure adherence. After the visit, the staff will use the urine sample for a rapid strip test to interpret adherence results. The staff will upload a picture of the test results to REDCap to send to the lab so that they can perform quality control checks. Participants’ urine samples will be used for 2 separate tests: one that measures TFV levels and another that measures FTC levels. Throughout the study period, participants (EPICC and mChoice) will be asked to track their PrEP adherence and sexual behavior in study mobile applications. Electronic health record (EHR) data (EPICC and mChoice Studies) will be collected every 6 months during the study period; variables will include PrEP prescription information, HIV testing, sexually transmitted infection (STI) testing, and results. Clinic site staff will complete medical record abstraction (MRA) using participants’ EHRs every 6 months while in the study. MRA will collect information about PrEP prescriptions, STI testing and results, and HIV testing and results. Table S1 in Multimedia Appendix 1 includes cohort measures.

A subset of EPICC project participants will also complete exit interviews. Questions will focus on understanding factors that influenced participants’ selection of PrEP regimens, changes, or discontinuations; perceptions of the counseling they received by providers at PrEP initiation and follow-up; and the receipt of tools or materials that influenced their choice and feasibility/acceptability of the HMP app. The mChoice project team will similarly conduct in-depth interviews with a subset of participants following completion of the intervention to explore experiences with PrEP, reasons for PrEP choices, and impressions of the mChoice intervention.

### Postcohort Provider Focus Group Discussions and Interviews

After the cohort is completed, the EPICC project team will conduct 6 virtual focus groups with PrEP providers, clinic staff, and study staff. The purpose of the focus groups will be to gather feedback on overall perceptions of the barriers and facilitators to education tool implementation within their clinical site. Prior to participating in the focus groups, participants will complete a survey that will collect their demographics, whether the provider can prescribe PrEP, and how long the provider has worked at their current clinic.

The mChoice project team will conduct in-depth interviews with participating providers following completion of the PrEP training modules and assessments. The research staff will ask participants about the implementation of the mChoice intervention, any long-term effects of the intervention, opinions of the provider training modules, and recommendations for future implementation of the mChoice intervention.

### Primary Outcome Measures

The primary outcome for provider training is PrEP familiarity, PrEP beliefs, and intentions to use PrEP measured at pre- and posttraining. The primary outcome for the cohort is PrEP adherence. For the EPICC project, adherence outcomes will be measured by using blood and will be tailored for oral PrEP modality (daily or 2-1-1) and categorized as protective (1) or not protective (0). The protective level of PrEP in the blood is defined as ≥4 doses taken per week, and the not-protective level is defined as <4 doses taken per week. Adherence outcomes for participants on CAB-LA will be determined by the timely administration of injections. The timely administration of the second injection is defined as within +/–1 week of the target date and within +/–2 weeks of the target date for subsequent injections. For the mChoice project, adherence outcomes, as measured by using urine, will be assessed by testing TFV and FTC levels in urine specimens collected from participants who report daily use of PrEP containing TFV and FTC. The mChoice project will also measure adherence by electronic medication monitoring via the CleverCap device, EHR data, and self-report.

### Secondary Outcome Measures

Secondary outcomes for provider training include the feasibility and acceptability of implementing provider training and barriers and facilitators impacting the implementation of new PrEP modalities in clinical practice measured through participant responses during the provider focus group discussions that will occur at the end of the cohort follow-up. The EPICC project cohort’s secondary outcome is persistence measures and will be based on (1) the participant’s self-report of currently taking PrEP (daily or 2-1-1) or having received the last shot of CAB-LA and (2) the participant having an active prescription for PrEP based on study records or drug levels associated with use within a 1-month window at 6 months, 12 months, and 18 months postenrollment. mChoice secondary outcomes will consist of sexual risk behaviors, HIV status, substance use, and outcomes by the initial PrEP regimen, as reported through follow-up assessments and EHR data.

### Statistical Analysis

Specific statistical analyses will be performed for the different projects. Next, we describe the EPICC and mChoice project analysis plans separately.

#### The EPICC Project

##### Sample Size and Power Calculations

The sample size for this study was determined based on feasibility after considering the intended analyses and multiple parameter estimates. With 400 participants, the minimum detectable difference in the PrEP adherence and persistence rates was 5.6%, assuming 15% loss to follow-up and a reference rate of 20%, at 0.80 power with a 1-sided test and 0.05 type I error. For baseline to postintervention analyses, simulation-based results indicated the minimum detectable difference from a baseline rate of 20% was approximately 9% (odds ratio 1.6), assuming again 15% loss to follow-up, at 0.80 power with a 1-sided test and 0.05 type I error [39].

##### Analysis Plan

Baseline participant characteristics will be described for the entire sample and disaggregated by regimen, site/region, ethnicity, level of HIV risk perception, substance use, PrEP experience (naive vs familiar), partner relationship status, and the other variables of interest included in Table S1 in Multimedia Appendix 1.

To determine any differences in discontinuation and nonadherence, we will use Cox proportional hazards to analyze (1) time to first discontinuation and (2) time to first nonadherence by regimen using PrEP adherence measures. To incorporate observations after the first discontinuation or after the first nonadherence, including allowing for the possibility of restarting on PrEP after discontinuation and for periods of nonadherence followed by a period of adherence, we will use a multilevel survival model by including a frailty term in the model to allow analysis of recurrent events [40]. We will follow the same analytic strategy for other dichotomous primary and secondary effectiveness and implementation outcomes. For the composite measure of number of prescriptions (primary implementation outcome), we will fit a multilevel linear or a generalized linear regression model, again using all the time point data available to assess the strength of the associations between the composite measure for the number of prescriptions and the predictor variables. To examine the trajectories of regimens, we will use generalized linear mixed models (GLMMs) and a survival model to assess the time to each regimen switch.

##### Provider Training

Mean scores for the pre- and postadministration of PrEP knowledge items, as well as the mean scores for motivational interviewing familiarity and comfort, will be evaluated for significance of difference using the nonparametric Wilcoxon signed-rank test for hypothesis testing of repeated measurements on a single sample [41].

#### The mChoice Project

##### Sample Size and Power Calculations

Sample size estimation was based on the number of individuals required in order to detect an odds ratio of 1.7 or greater, based on a previous intervention study [42], in the primary outcome measures (PrEP adherence and persistence) before and after the intervention. We used a GLMM [43] with 80% power and a 2-sided test of .05 significance. We estimated the power and sample size by simulating responses based on the following assumptions: 20% attrition postintervention follow-up, an intraclass correlation coefficient of 0.2 across sites, and correlations in the range of 0.3-0.6 of participants’ outcomes at different time points [43]. With these conditions, a sample size of 400 was needed [43].

##### Primary Outcome

A GLMM, also called an individual growth model and a multilevel model, with an appropriate link function will be used to compare the pre- and postintervention difference for each outcome. The GLMM allows different trajectories for each participant, and this method is appropriate to compare outcome changes after the implementation of the intervention, with the control of baseline values. Analyses will be conducted for the full sample and by study location (New York City and Birmingham) separately [44].

##### Secondary Outcomes

Similar GLMMs will be used for analyzing secondary outcomes. We will conduct a multigroup comparison in pre- and postintervention differences (the difference-in-difference analysis) using a GLMM by adding the group variable and the group × intervention status interaction (pre- and postintervention) in the GLMM described before. Because the PrEP regimen cannot be randomized, we will use the propensity score method [45] to reduce the between-group bias. We will also examine factors that are associated with the length of time that participants take to change their regimens or associated with the instantaneous rate of change of regimen. Since we will know the date of change of the PrEP regimen (from the EHR data), we will apply a Cox proportion hazard ratio model with time-varying covariates (eg, sexual activity, insurance, side effect) to examine the time to change the regimen.

##### Provider Training

Mean scale scores for the pre- and postadministration of PrEP knowledge items, as well as the mean time with PrEP patients, will be evaluated for significance of differences using the nonparametric Wilcoxon signed-rank test for hypothesis testing of repeated measurements on a single sample [41]. Categorical data for assessing differences in the proportion of participants in agreement with individual items before and after participating in the knowledge module will be analyzed using the McNemar test of marginal homogeneity [41].

## Results

The EPICC project formative work to develop evidence-based tools (Maragh-Bass et al, unpublished data, March 2025) was completed in April 2023 [17]. Provider training enrollment began in January 2024 and was completed in August 2024. Cohort enrollment began in April 2024. As of October 2024, 40 participants were enrolled in the EPICC project cohort. Cohort enrollment is expected to be completed in September 2025, with the final results anticipated in early 2027. Provider focus groups are expected to begin in April 2026, with the final results expected in early 2027.

The mChoice project formative work to develop and evaluate evidence-based tools was completed in January 2024 (Kay et al, unpublished data, March 2025) [18,46]. Provider training enrollment began in June 2024 and is ongoing. Cohort enrollment began in July 2024. As of October 2024, 18 participants were enrolled in the mChoice project cohort. Cohort enrollment is expected to be completed in July 2025. The final results are expected in late 2027.

## Discussion

### Overview

Posttraining, we anticipate providers will increase competence in using EBTs and providing PrEP support services. We also anticipate participants in the cohort will increase PrEP adherence and persistence. Given the changing PrEP landscape and the availability of new options and formulations, the implementation of provider education and tools to maximize uptake and adherence within their patient populations is needed. By delivering culturally competent and interactive provider training on PrEP options, the study will help providers counsel and guide participants on the effective and safe use of PrEP. The digital health tools created will support participant adherence and help them optimize the prevention benefits of their chosen PrEP regimen. Through the longitudinal, cohort design, the PrEP Choice study will provide real-world data about PrEP use that will be critical for informing future guidelines and tools.

### Limitations

This research is limited in its design as a cohort study and not a randomized controlled trial. However, early work to harmonize measures and outcomes across the 2 projects is expected to allow for informative descriptions and the possibility for both pooled and separate analyses, as well as comparison across the entire study. Although multiple study sites across the United States are included, PrEP Choice is not a nationally representative study and results will not be generalizable.

### Conclusion

A multitude of efforts exist to make PrEP more available for people at risk for acquiring HIV infection, especially YMSM. Activities to increase PrEP uptake need to be accompanied by research that assesses its real-world use and identifies strategies to support and maximize its benefits. In addition to improving our understanding of how those at increased risk for acquiring HIV infection use PrEP, the PrEP Choice study will support the design and testing of informed interventions to support PrEP users’ adherence and persistence, in addition to initiation.

## Appendix

Multimedia Appendix 1 Cohort measures for the Expanding Pre-Exposure Prophylaxis (PrEP) in Communities of Color (EPICC) and mChoice Studies.

## Abbreviations

CAB-LA: long-acting injectable cabotegravir
CASI: computer-assisted self-interview
CDC: Centers for Disease Control and Prevention
CMT: Compliance Meds Technologies
DBS: dried blood spot
EBT: evidence-based provider and patient education and support tool
EHR: electronic health record
EPICC: Expanding Pre-Exposure Prophylaxis (PrEP) in Communities of Color
F/TAF: tenofovir alafenamide
FTC: emtricitabine
FTC-TP: emtricitabine-triphosphate
F/TDF: tenofovir disoproxil fumarate
GLMM: generalized linear mixed model
HMP: HealthMpowerment
MI: motivational interviewing
MRA: medical record abstraction
MSM: men who have sex with men
PEP: postexposure prophylaxis
PrEP: pre-exposure prophylaxis
STI: sexually transmitted infection
TFV: tenofovir
TFV-DP: tenofovir-diphosphate
TMI: tailored motivational interviewing
YMSM: young men who have sex with men

## Data Availability

The datasets generated and analyzed during this study will be available from the corresponding author upon reasonable request.
