# Supplementary material for: Developing and Implementing Provider-Training and Evidence-Based Tools to Support Pre-exposure Prophylaxis (PrEP) Decision-Making and Increase PrEP Adherence Among Young Men Who Have Sex With Men: Protocol for the PrEP Choice Longitudinal Cohort Study
Source: JMIR Res Protoc. 2025 Mar 20;14:e64186. doi: 10.2196/64186 (PMC11969124; doi:10.2196/64186)
Supplement: Multimedia Appendix 1 [file resprot_v14i1e64186_app1.docx]

**Table S1.** Cohort measures for the Expanding Pre-Exposure Prophylaxis (PrEP) in Communities of Color (EPICC) and mChoice Studies.

| Domain and measure | | Baseline | 3 months | 6 months | 9 months | 12 months | 15 months | 18 months |
| --- | --- | --- | --- | --- | --- | --- | --- | --- |
| **PrEP adherence and persistence** | | | | | | | | |
|  | App-based tracking diaries^a,b^: self-report PrEP doses taken or missed each day, record of doses taken or missed through CleverCap device and app, record of potential exposure events, and 2-1-1 doses taken. | ✓ | ✓ | ✓ | ✓ | ✓ | ✓ | ✓ |
|  | Home-based specimen collection via dried blood spots (DBSs)^c^: concentrations of emtricitabine (FTC) and tenofovir (TFV) | ✓ |  | ✓ |  | ✓ |  | ✓ |
|  | Clinic-based specimen collection via urine^d^: concentrations of FTC and TFV | ✓ | ✓ | ✓ | ✓ | ✓ | ✓ | ✓ |
|  | Self-report PrEP adherence^e^: number of doses taken (adjusted for dosing strategy) assessed using the Brief Medication Questionnaire Visual Analogue Scale [1,2] | ✓ | ✓ | ✓ | ✓ | ✓ | ✓ | ✓ |
|  | Adherence to refills and clinic visits^f^: PrEP prescriptions issued, visits attended/missed | ✓ |  | ✓ |  | ✓ |  | ✓ |
| **PrEP decision-making (uptake, choice, switches)** | | | | | | | | |
|  | App-based tracking diaries^a,b^: tracking of PrEP formulations used | ✓ | ✓ | ✓ | ✓ | ✓ | ✓ | ✓ |
|  | PrEP decision-making^e^: reasons for PrEP use and choice, counseling received by providers at PrEP initiation and follow-up, receipt of tools or materials that influence choice, PrEP confidence [3] | ✓ | ✓ | ✓ | ✓ | ✓ | ✓ | ✓ |
|  | PrEP formulations prescribed and received^f^: electronic health record (EHR) prescriptions issued data, visits attended/missed | ✓ |  | ✓ |  | ✓ |  | ✓ |
|  | PrEP discontinuation^e^: reasons for PrEP changes and discontinuations [4] and sexual behavior after change and discontinuations | ✓ | ✓ | ✓ | ✓ | ✓ | ✓ | ✓ |
|  | PrEP stigma^e^ and positive attitudes [5] | ✓ |  | ✓ |  | ✓ |  | ✓ |
|  | PrEP knowledge, awareness, and communication^e^ | ✓ |  | ✓ |  | ✓ |  | ✓ |
| **Sexual Risk Behaviors** | | | | | | | | |
|  | App-based tracking diaries^a,b^: sexual behavior each day as relevant to activities | ✓ | ✓ | ✓ | ✓ | ✓ | ✓ | ✓ |
|  | Sexual risk over the past 3 months^e^: number of condomless anal sex encounters by partner, partner characteristics | ✓ | ✓ | ✓ | ✓ | ✓ | ✓ | ✓ |
|  | Sexually transmitted infection (STI) testing and diagnosis^f^: STI tests and results, including oral, urethral, and rectal gonorrhea; oral urethral and rectal chlamydia; syphilis; any other STI tests and diagnoses | ✓ |  | ✓ |  | ✓ |  | ✓ |
| **Additional variables of interest** | | | | | | | | |
|  | Demographics^e^: demographics, sexual identity, employment, income, housing, zip code, health insurance | ✓ |  | ✓ |  | ✓ |  | ✓ |
|  | Provider/patient processes of care^e^: provider-patient communication | ✓ |  | ✓ |  | ✓ |  | ✓ |
|  | Social support^e^ and isolation [6] | ✓ |  | ✓ |  | ✓ |  | ✓ |
|  | Perceived discrimination (race/ethnicity and sexual identity)^e^: Everyday Discrimination Scale [7] perceived discrimination based on race/ethnicity and sexual identity | ✓ |  | ✓ |  | ✓ |  | ✓ |
|  | Mental health (depression, anxiety)^e^: 9-item Patient Health Questionnaire (PHQ-9) [8], 7-item Generalized Anxiety Disorder (GAD-7) [9] | ✓ |  | ✓ |  | ✓ |  | ✓ |
|  | Substance use^e^: World Health Organization (WHO) Alcohol, Smoking & Substance Involvement Screening Test (ASSIST) [10] | ✓ |  | ✓ |  | ✓ |  | ✓ |
|  | Intervention acceptability^e^: access to technology [11], EPICC+ acceptability [12] | ✓ |  |  | ✓ |  |  | ✓ |

^a^EPICC app: Participants use the health tracker to record medication adherence and sexual activity.

^b^mChoice app: Participants use the Dashboard page to record medication adherence and sexual activity. Monthly adherence data for daily PrEP users will be displayed on the My Stats page.

^c^DBS: Participants complete an at-home DBS kit at baseline and every 6 months while in the study to assess for levels of tenofovir-diphosphate (TFV-DP) and emtricitabine-triphosphate (FTC-TP).

^d^Urine: Participants who use daily oral PrEP provide a urine sample at baseline and every 3 months while in the study to assess levels of TFV and FTC.

^e^Computer-assisted survey interviewing (CASI): Participants will complete a CASI every 3 months while in the study. Surveys will be hosted on REDCap. EPICC surveys will be distributed through emails and links available within the study app.

^f^EHRs: Data will be abstracted from participants’ EHRs at baseline and every 6 months while in the study.

## References

1. Wewers ME, Lowe NK. A critical review of visual analogue scales in the measurement of clinical phenomena. *Res Nurs Health* 1990 Aug; 13(4):227-236.
2. Finitsis DJ, Pellowski JA, Huedo-Medina TB, Fox MC, Kalichman SC. Visual analogue scale (VAS) measurement of antiretroviral adherence in people living with HIV (PLWH): a meta-analysis. *J Behav Med* 2016 Dec; 39(6):1043-1055.
3. Budhwani H, Yigit İ, Maragh-Bass AC, Rainer CB, Claude K, Muessig KE, Hightow-Weidman LB. Validation of HIV pre-exposure prophylaxis (PrEP) medication scales with youth on PrEP: PrEP confidence scale and PrEP difficulties scale. *AIDS Patient Care STDS* 2022 Nov; 36(11):443-450.
4. Morgan E, Ryan DT, Newcomb ME, Mustanski B. High rate of discontinuation may diminish PrEP coverage among young men who have sex with men. *AIDS Behav* 2018 Nov; 22(11):3645-3648.
5. Budhwani H, Yiğit İ, Maragh-Bass AC, Rainer CB, Claude K, Muessig KE, Hightow-Weidman LB. Development and validation of the youth pre-exposure prophylaxis (PrEP) stigma scale. *AIDS Behav* 2023 Mar; 27(3):929-938.
6. Hahn EA, DeWalt DA, Bode RK, Garcia SF, DeVellis RF, Correia H, Cella D, PROMIS Cooperative Group. New English and Spanish social health measures will facilitate evaluating health determinants. *Health Psychol* 2014 May; 33(5):490-499.
7. Krieger N, Smith K, Naishadham D, Hartman C, Barbeau EM. Experiences of discrimination: validity and reliability of a self-report measure for population health research on racism and health. *Soc Sci Med* 2005 Oct; 61(7):1576-1596.
8. Kroenke K, Spitzer RL, Williams JB. The PHQ-9: validity of a brief depression severity measure. *J Gen Intern Med* 2001 Sept; 16(9):606-613.
9. Spitzer RL, Kroenke K, Williams JBW, Löwe B. A brief measure for assessing generalized anxiety disorder: the GAD-7. *Arch Intern Med* 2006 May 22; 166(10):1092-1097.
10. WHO ASSIST Working Group. The Alcohol, Smoking and Substance Involvement Screening Test (ASSIST): development, reliability and feasibility. *Addiction* 2002 Sept; 97(9):1183-1194.
11. Norman CD, Skinner HA. eHEALS: the eHealth Literacy Scale. *J Med Internet Res* 2006 Nov 14; 8(4):e27.
12. Chen E, Moracco KE, Kainz K, Muessig KE, Tate DF. Developing and validating a new scale to measure the acceptability of health apps among adolescents. *Digit Health* 2022; 8:20552076211067660.
